# Functional Components from the Liquid Fermentation of Edible and Medicinal Fungi and Their Food Applications in China

**DOI:** 10.3390/foods12102086

**Published:** 2023-05-22

**Authors:** Meng-Qiu Yan, Jie Feng, Yan-Fang Liu, Dian-Ming Hu, Jing-Song Zhang

**Affiliations:** 1 Key Laboratory of Edible Fungi Resources and Utilization (South), Key Laboratory of Agricultural Genetics and Breeding of Shanghai, Institute of Edible Fungi, Shanghai Academy of Agricultural Sciences, National Engineering Research Center of Edible Fungi, Ministry of Agriculture of P. R. China, Shanghai 201403, China; yanmengqiu@sina.com (M.-Q.Y.); sytufengjie@163.com (J.F.); aliu-1980@163.com (Y.-F.L.); 2 Bioengineering and Technological Research Centre for Edible and Medicinal Fungi, Jiangxi Agricultural University, Nanchang 330045, China; 3 Jiangxi Key Laboratory for Conservation and Utilization of Fungal Resources, Jiangxi Agricultural University, Nanchang 330045, China

**Keywords:** edible and medicinal fungi, liquid fermentation, active ingredient, functional food, fruiting body, research progress

## Abstract

Functional raw materials rich in various effective nutrients and active ingredients that are of stable quality can be obtained from the liquid fermentation of edible and medicinal fungi. In this review, we systematically summarize the main findings of this comparative study that compared the components and efficacy of liquid fermented products from edible and medicinal fungi with those from cultivated fruiting bodies. Additionally, we present the methods used in the study to obtain and analyze the liquid fermented products. The application of these liquid fermented products in the food industry is also discussed. With the potential breakthrough of liquid fermentation technology and the continued development of these products, our findings can serve as a reference for further utilization of liquid fermented products derived from edible and medicinal fungi. Further exploration of liquid fermentation technology is necessary to optimize the production of functional components from edible and medicinal fungi, and to enhance their bioactivity and safety. Investigation of the potential synergistic effects of combining liquid fermented products with other food ingredients is also necessary to enhance their nutritional values and health benefits.

## 1. Introduction

Edible and medicinal fungi usually refer to large fungi that have been used as a pharmaceutical or food source. There are more than 270 species of edible and medicinal fungi recorded in China [1,2]. The use of edible and medicinal fungi can be traced back to thousands of years when wild fungi were picked and eaten. With the development of modern science and technology, the artificial cultivation of fruiting bodies and fermentation of mycelium has appeared in the production methods of edible and medicinal fungi and at present, more than 60 kinds of edible and medicinal fungi are cultivated artificially. Among them, more than 20 species are produced commercially such as *Lentinus edodes*, *Flammulina velutipes*, *Agaricus bisporus*, *Pleurotus ostreatus*, *Auricularia auricula*, *Pleurotus eryngii*, *Ganoderma lucidum*, *Hericium erinaceus*, etc. [3,4]. In recent years, the technology of liquid fermentation for edible and medicinal fungi has been rapidly developing. Compared with traditional artificial cultivation and solid fermentation technology, liquid fermentation has the advantages of a short production cycle, high production efficiency, stable product quality and easy separation of products, which can effectively improve the production efficiency of target products and can be widely used in industrial production [5,6].

Liquid fermentation (also known as submerged culture) is a technology in which the nutrients needed for mycelial growth are prepared in a liquid culture medium, and then mycelium is inoculated for further culture. In the 1940s, Elmer and Caden, two bioengineering experts from the University of Virginia (USA), first designed a bioreactor for microbial culture [7], this technology was then widely used in the industrial production of antibiotics, thus initiating the liquid fermentation of edible and medicinal fungi. In 1948, Humfeld in America first used liquid fermentation technology to cultivate *Agaricus bisporus* mycelium [8]. Since 1958, China has been researching the liquid fermentation of *Agaricus bisporus* and *Pleurotus ostreatus*. Since 1963, when the industrial production of the liquid fermentation of *Morchella esculenta* was first developed, liquid fermentation technology has been applied to the production of edible and medicinal fungi on a large scale, and the liquid fermentation culture of a series of edible and medicinal fungi, such as *Ganoderma lucidum*, *Armillaria mellea*, *Tremella fuciformis*, *Lentinus edodes*, *Cordyceps sinensis*, *Pleurotus ostreatus*, *Auricularia auricula*, *Coriolus versicolor*, *Anluo umbrella*, *Ergot fungi*, *Polyporus umbellatus*, *Poria cocos*, *Flammulina velutipes*, *Hericium erinaceus*, *Termite umbrella*, *Volvariella volvacea and Dictyophora indusiate,* has been developed [1].

The liquid fermentation of edible and medicinal fungi has been widely applied in the fields of medicine, food and feed [9,10,11,12]. Liquid fermented products can be divided into mycelium and extracellular fluid that mainly contain amino acids, vitamins, polysaccharides, triterpenes, proteins, alkaloids, glycosides, sterols, flavonoids and antibiotics and these functional components are usually derived from medicinal fungi. Polysaccharides, triterpenes, flavonoids, alkaloids and proteins have been extensively studied. They have many biological activities such as immunoregulation, anti-tumor, anti-virus, antioxidant, anti- aging, hypoglycemic, lipid-lowering, etc. For example, *Hericium erinaceus* tablets that have entered the market are a kind of traditional Chinese medicine made from liquid fermented *Hericium erinaceus* mycelium extract. This is used to strengthen the body’s resistance and protect the stomach, and has been clinically used to treat digestive tract diseases such as gastric peptic ulcer, duodenal ulcer, chronic gastritis and atrophic gastritis [13].

Currently, people’s demands for food are gradually developing from “enough food” to “good food” and then to “healthy food” [14,15]. Fermented products from edible and medicinal fungi are such food resources with health care functions that have preventive and therapeutic effects on certain diseases. The fermentation products contain many active components such as amino acids, vitamins, enzymes, polysaccharides, alkaloids, glycosides, sterols, flavonoids and antibiotics. Furthermore, fungal polysaccharides have many biological activities such as anti-tumor, anti-virus, anti-aging, hypoglycemic and lipid-lowering effects as well as immunity improving effects [16,17,18]. Therefore, liquid fermented products of edible and medicinal fungi have a wide application prospect in the field of functional food.

Based on the above analysis, this paper systematically summarizes the main components and efficacy of liquid fermented products from edible and medicinal fungi, and compares them with cultivated fruiting bodies. The application of the related liquid fermented products in the food industry are also summarized in order to provide references for the application and popularization of the liquid fermentation of edible and medicinal fungi.

## 2. Main Components and Efficacy of the Liquid Fermentation of Edible and Medicinal Fungi

During the mycelial growth of edible and medicinal fungi, the metabolism will secrete a large amount of nutrients and active ingredients that can be extracted from both the mycelium and extracellular fluid. These nutrients and active ingredients contain many active substances such as polysaccharides, triterpenes, proteins, amino acids, vitamins, alkaloids, glycosides, sterols, flavonoids and antibiotics. Among these, triterpenes, polysaccharides and proteins are the most widely studied. They have immunoregulatory, anti-tumor, antioxidant, anti-viral, hypoglycemic and lipid-lowering properties.

### 2.1. Main Components of the Liquid Fermentation of Edible and Medicinal Fungi

#### 2.1.1. Polysaccharides

The liquid fermentation of polysaccharides from edible and medicinal fungi can be divided into intracellular polysaccharides (IPSs) and extracellular polysaccharides (EPSs), or they can be divided into glucans and heteropolysaccharides according to the sugar composition. Glucans are composed of single glucose, which can be further divided into α configuration and β configuration in space conformation, and the active glucans in edible and medicinal fungi are mainly of the β configuration; heteropolysaccharides are composed of a variety of monosaccharides with different molar ratios, and the most common monosaccharide composition of mycelium from edible and medicinal fungi is fructose, xylose, mannose and ribose [19].

Fermented polysaccharides have anti-tumor, immunity improvement and anti-oxidative functions. Jing et al. [20] isolated two exopolysaccharide components with different molecular weights from *Pleurotus eryngii* that have antioxidant and anti-tumor effects; among them, the low molecular weight exopolysaccharides with better solubility has greater activity. Assis et al. [21] found that the EPSs from *Pleurotus ostreatus* have anti-tumor activities. Luo et al. [22] separated and purified the extracellular homogeneous polysaccharide EPS-2-1 and intracellular polysaccharide IPS-2-1 from liquid fermented *Ganoderma lucidum*, and the polysaccharides had the ability to scavenge the DPPH and hydroxyl radicals, while EPS-2-1 had better antioxidant ability. Du et al. [23] isolated an exopolysaccharide with a molecular weight of 2900 kDa from the mycelium extracted from the submerged culture of *Schizophyllum schizophyllum*. Polysaccharides can inhibit the expression of nitric oxide synthase induced by LPSs (lipopolysaccharides) in vitro and have some anti-inflammatory activities.

#### 2.1.2. Triterpenoids

Triterpenes from edible and medicinal fungi are lanosterane derivatives that can be divided into C24, C27 and C30 according to the number of carbon atoms. According to the structure, they can be divided into tetracyclic and pentacyclic triterpenes. According to the different functional groups and side chains, triterpenes can be divided into *Ganoderma lucidum* acid, *Ganoderma lucidum* alcohol, *Ganoderma lucidum* aldehyde and *Ganoderma lucidum* lactone. *Ganoderma lucidum* acid and *Ganoderma lucidum* alcohol are the most abundant triterpenes; according to polarity, triterpenes can be divided into medium polar triterpenes and low polar triterpenes [24].

Anti-tumor and antibacterial activities of triterpenoids from mycelium are their main activities. Zhu et al. [18] isolated four triterpenoids from the liquid fermentation of *Ganoderma lucidum* mycelium through silica gel column chromatography, reversed-phase column chromatography and methanol recrystallization; the isolated triterpenoids inhibited the proliferation of tumor cells K562 and L1210. Cai et al. [25] investigated the antibacterial effects of total triterpenes from fermented *Phellinus linteus*. The results showed that the total triterpenes from *Phellinus linteus* had inhibitory effects on *Escherichia coli* Y35, *Staphylococcus aureus*, *Bacillus subtilis* and *Bacillus thuringiensis*; the results of anti-tumor tests showed that the extract could strongly inhibit the proliferation of colon cancer cells CaCO_2_, and a flow cytometry analysis showed that it blocked the cell cycle and induced the programmed apoptosis of CaCO_2_. The antioxidant activity of 300 ug/mL total triterpene extract on hydroxyl radical, superoxide anion, DPPH radical and ABTS was over 90%. Zhou et al. [26] discovered five triterpenes with lipoxygenase inhibition from triterpene extracts using high performance liquid chromatography–electrospray ionization mass spectrometry (HPLC-ESI-MS).

#### 2.1.3. Proteins

Proteins are abundant in edible and medicinal fungi, their content is generally between 20 and 50% (dry weight), and the proportion of essential amino acids is reasonable [27]. Kurbanoglu et al. [28] studied the submerged culture of *Agaricus bisporus* in ram horn hydrolysate, and its protein concentration was 47%; an amino acid analysis showed that *Agaricus bisporus* protein contained many essential amino acids and it had high nutritional value. Rahgo [29] optimized the fermentation medium of a new variety of *Morchella esculenta* obtained in northern Iran, and the protein content reached 38% (including 28.7% essential amino acids).

There are many nutritional evaluation indexes for edible fungal proteins, such as biological evaluations, non-biological evaluations, comprehensive evaluations based on the essential amino acid composition, digestibility, etc. Gang et al. [30] evaluated the proteins and amino acids in liquid fermented *Cordyceps militaris*, and the protein content was 21.1%. The amino acid score AAS, chemical score CS, essential amino acid index EAAI, biological value BV, nutritional index NI and amino acid correlation ratio SRCAA were recorded at 62.41, 38.74, 88.37, 84.63, 18.61 and 25.57, respectively. The protein content was 2.52% higher than the model value of the total essential amino acids of eggs and was 45.57% higher than the FAO/WHO model value.

### 2.2. Comparison of the Main Components of Edible and Medicinal Fungi in Liquid Fermentation and Fruiting Body Cultivation

The main active components isolated from the fruiting bodies of edible and medicinal fungi are polysaccharides, triterpenes, proteins, alkaloids, sterols, etc. These active substances exhibit anti-tumor, immunoregulatory, antioxidant, hypoglycemic and lipid-lowering effects [31,32]. The active components that are separated during liquid fermentation are basically the same as those from fruiting bodies, and the content of some important active components, such as polysaccharides from liquid fermentation, is higher than that from the fruiting bodies. Some studies also proved that there are some components in fruiting bodies that are not found in mycelium such as some triterpenoids. Liu et al. [17] compared the polysaccharide, triterpene, alditol and nucleoside contents between *Ganoderma lucidum* fruiting body and the liquid fermented mycelium. The results showed that the polysaccharide content in mycelium was 1.54%, which was significantly higher than that in the fruiting body (0.79–0.87%); the molecular weight distribution of the mycelium polysaccharides was 2.31 × 10^5^ Da, while that of the fruiting body was 3.27 × 10^4^–1.95 × 10^6^ Da. They both contained arabinitol and mannitol, and the mycelium also contained a small amount of erythritol. The nucleoside content in mycelium was lower than that in the fruiting body, but the cytidine, guanosine and adenosine contents were higher in the fermented mycelium. Ten triterpenes were detected in the fruiting body, but only *Ganoderma lucidum* acid A and *G. lucidum* keto-triol were detected in the mycelium with greater content. Zhang [33] also found that the fermented mycelium and fruiting body of *Ganoderma lucidum* had some of the same components, but their contents were different; the crude polysaccharide and polysaccharide contents of mycelium were 2.26 times and 3.5 times higher than those in the fruiting body, and the crude protein content was 2.47 times higher than that in the fruiting body. The essential amino acids of the fruiting body accounted for 58.4% of the total amino acids, while the proportion in the mycelium was 45.2%.

Some studies have shown that there may be different results in the evaluation of proteins and amino acids in edible and medicinal fungi using different evaluation criteria for the nutritional value. Xi et al. [34] evaluated the protein of the lotus leaf fruiting body and mycelium, and found that the nutritional values of the fruiting body and mycelium were slightly different; For example, the amino acid composition of the proteins from the fruiting body was closer to that of standard egg white, but the amino acid composition of the proteins from the mycelium was closer to the FAO/WHO model. However, the nutritional value of the mycelium was better than that of the fruiting body considering the nutritional balance, and the proportion of essential amino acids in the fruiting body was 40.2%, which was higher than that in mycelium (35.3%). Some amino acids in mycelium, such as lysine and leucine, were higher than those in the fruiting body, while others were close to those in the fruiting body. The protein content in mycelium fermentation broth was low, only six amino acids were found including valine and tyrosine. The chemical score and amino acid score of mycelium were 73.4 and 80.2, respectively, which were significantly higher than those of the fruiting body: 45.4 and 57.8. The essential amino acid index of the fruiting body was 76.8 and that of mycelium was 70.7. The nutritional value of mycelium proteins was slightly lower than that of the fruiting body, but the nutritional index of the fruiting body was 16.4, which was lower than that of mycelium: 20.0.

Table 1 summarizes the nutritional components of the mycelium and fruiting bodies of edible and medicinal fungi observed in the literature, and the results show that there is little difference in the nutritional components between the fruiting bodies and mycelium. In some species, the total sugar, protein and other nutrients in mycelium are higher than that in the fruiting bodies. while in other species, the outcome is quite different. However, the crude fiber of mycelium from edible and medicinal fungi listed in Table 1 are all lower than that of the fruiting bodies, which may suggest that mycelium may be more beneficial for human absorption and utilization.

### 2.3. Biological Activity of the Liquid Fermented Products of Edible and Medicinal Fungi

Liquid fermented mycelium and extracellular extracts of edible and medicinal fungi have anti-tumor, immunoregulatory, antioxidant, antibacterial and antiviral activities.

#### 2.3.1. Anti-Tumor

The anti-tumor effect of edible and medicinal fungi can be manifested in the prevention of tumor occurrence and the killing effect of the produced tumor. Gao [42] investigated the anti-tumor activity of ethanol extract from the fermented mycelium of *Agaricus blazei* in vivo and in vitro. The results showed that the semi-inhibitory concentration of the extract on the human hepatoma cell Bel-7402 was 1507 μg/mL, and it also had a certain effect on the inhibition of the S180 tumor in tumor-bearing mice with a life prolongation rate of 52.94%. Huang et al. [43] found that *Ganoderma pine* mycelium and fermentation broth could inhibit the proliferation of H22 liver ascites tumor cells by improving the immune function in mice. Fijakowska et al. [44] found that indole, phenol and sterol compounds contained in the mycelium of *Laminophyllum* hadcertain antioxidant and inhibitory proliferation effects on A549 lung cancer, DU145 prostate cancer and A376 melanoma cells. Yu [45] investigated the inhibition rate of *Tricholoma matsutake* crude polysaccharides on three kinds of human tumor cells. The results showed that the inhibition rates of 10 mg/mL polysaccharides on melanoma B16, liver cancer cell SMMC7721 and cervical cancer cell Hela were 63.54%, 62.43% and 57.81%, respectively. Zhu et al. [18] isolated four triterpenoids from the liquid fermented mycelium of *Ganoderma lucidum* and found that they could effectively inhibit the proliferation of K562 and L1210 tumor cells. Li [46] investigated the anti-tumor effect of *Ganoderma lucidum* polysaccharides and tetracyclic triterpene acid. The results showed that the average inhibition rate of a single polysaccharide was 51.2%, and that of mixed samples reached 68.0%. The results provide a reference for the effective compound use of fermented products.

#### 2.3.2. Immunoregulation

The immunomodulatory effect of polysaccharides from edible and medicinal fungi is the basis of other functions such as anti-tumor effect. SPG, an intracellular homogeneous polysaccharide of Schizophyllum was isolated by Li [47], and it was determined that it could restore the proliferation response of spleen lymphocytes in aged mice and could ameliorate the delayed skin allergic reaction of mice induced by DNBC. It also increased the level of mouse splenic orifice forming cells induced by sheep red blood cells in adult mice and improved the cellular and humoral immune functions of aged mice. Li [48] isolated three kinds of homogeneous polysaccharides from the liquid fermented mycelium of *Umbrella dinghuensis* and studied their immune activity in the mouse model. The results showed that the three polysaccharides could restore the immunity induced by cyclophosphamide and at the same time improve the spleen index, ear swelling from a delayed type of hypersensitivity and lysozyme activity in spleen and serum at the same time. Carrieri et al. [49] extracted a water-soluble heteropolysaccharide GLP-3 from *Ganoderma lucidum* mycelium, and it was determined that it could be recognized by the toll-like receptor, and play an immunomodulatory role by activating MAPKS, PI3K)/Akt and NF-κ B signaling pathways in macrophage RAW264.7.

#### 2.3.3. Antioxidant

The mycelium and extracellular fluid extracts of various edible and medicinal fungi have antioxidant effects. Hu [50] purified an irregular linear homogeneous polysaccharide PHEB with a molecular weight of 36.1 kDa from the fermented mycelium of *Hericium erinaceus*, which can ameliorate neuronal damage in the brain of Alzheimer’s disease mice. Li [48] purified three polysaccharide components from the fermented mycelium of *Umbrella dinghuensis*, which have antioxidant activities for scavenging hydrogen peroxide, DPPH radical and hydroxyl radical, and chelating ferrous ions (Fe^2+^). Liu [51] found that the scavenging activity of the exopolysaccharides of *Inonotus obliquus* on the DPPH and hydroxyl radicals was better than that of mycelial polysaccharides. Zhang et al. [52] extracted the mycelial protein of *Morchella esculenta* through alkali dissolution and acid precipitation, and the protein was determined to have antioxidant properties for scavenging hydrogen peroxide, DPPH radical and hydroxyl radical, and the IC50 values of the total antioxidant capacity and reducing power were 6.93 mg/mL and 4.24 mg/mL, respectively.

#### 2.3.4. Antibacterial and Antiviral

Studies have shown that many kinds of edible and medicinal fungi fermentation broth sand their extracts have inhibitory effects on common bacteria. Dou et al. [53] investigated the antibacterial effect of liquid fermentation broth from seven common edible and medicinal fungi, such as *Lentinus edodes* and *Coprinus comatus*, on three drug-resistant fungi: *Streptococcus pneumoniae*, *Staphylococcus aureus* and *Escherichia coli*. The results showed that the fermentation broth of edible and medicinal fungi had good antibacterial activity against *Staphylococcus aureus* The diameter of the antibacterial zone of *Flammulina velutipes* fermentation broth was 6.733 mm, and the antibacterial rate was 58.586%. The bacteriostatic activity of the fermented broth of edible and medicinal fungi against *Streptococcus pneumoniae* was low, the diameter of the bacteriostatic zone of *Coprinus comatus* fermented broth was 4.433 mm, and the bacteriostatic rate was 30.370%. The inhibition rate of the tested edible and medicinal fermentation broth against *Escherichia coli* was less than 10% which showed that the inhibition effect is not good.

### 2.4. Comparison of the Active Substances in Liquid Fermented Mycelium and the Cultivated Fruiting Body of Edible and Medicinal Fungi

The difference in nutritional composition or proportion between the fruiting body and mycelium of edible and medicinal fungi leads to different efficacies or different levels of efficacy, and this provides a basis for enterprises to select suitable products for application. Due to the different types of products, such as the different varieties, different cultivation or fermentation conditions, the existing studies on the efficacy of fermentation products and cultivation products of the same variety are limited, and these are summarized in Table 2. Yu et al. [54] proved that *Ganoderma lucidum* (G0109) mycelial polysaccharides could stimulate more NO production by macrophage Raw264.7 than the fruiting body polysaccharides, especially at low doses, and this may be related to the differences in the polysaccharide content, molecular weight, monosaccharide composition and configuration. Cai et al. [55] investigated the antioxidant activity of *Ganoderma lucidum* fruiting body and mycelium, and found that the water extract of the fruiting body had the strongest scavenging ability for the hydroxyl radical, and the water extract of mycelium had the strongest scavenging ability for hydrogen peroxide. Zhang et al. [56] investigated the differences in composition and activity between the fermented mycelium and the fruiting body of *Phellinus linteus* in four extraction phases: petroleum ether, chloroform, ethyl acetate and n-butanol. The results showed that the content of total flavonoids in the ethanol extract of mycelium was higher than that in the fruiting body, and the antioxidant activity from each phase of mycelium was higher than that of the fruiting body, while the anti-tumor activity of the fruiting body was higher than that of mycelium, suggesting that there was a significant correlation between the antioxidant activity and total flavonoids content. Chen et al. [57] optimized the alkali extraction and acid precipitation extraction method of *Pleurotus ostreatus* mycelium protein. Under the best extraction conditions, the extraction rate of mycelium protein reached 39.02%, and its emulsifying property was 48%, which was higher than that of the fruiting body protein (6%), but the foaming property of the fruiting body protein was relatively better. This conclusion also provided reference for the differential application of *Pleurotus ostreatus* mycelium protein and the fruiting body protein.

## 3. Application of Liquid Fermented Products from Edible and Medicinal Fungi in the Food Industry

Liquid fermented products from edible and medicinal mushrooms have wide application prospects in the food industry such as producing flavored food and beverages with its special taste, developing functional foods and health products with its active ingredients and producing healthy imitation meat products with its high protein and low-fat characteristics. The applications of them in food were summarized in Table 3.

### 3.1. General Food Fields

#### 3.1.1. Flavor in Food

The special aroma and flavor of edible fungi can enhance the flavor of food. Their unique flavor mainly comes from C8 volatile compounds and some unsaturated fatty acids such as oleic acid, linoleic acid, linolenic acid, palmitic acid and palm [59,60]. Through submerged fermentation, the products with high quality taste and flavor can be extracted from mycelium to meet the industrial production demands. Wu [61] produced *Tricholoma mongolicum* sauce with liquid fermented mycelium from *Tricholoma mongolicum* as the raw material and supplemented it with salt, sucrose, monosodium glutamate and citric acid. Fu [62] developed Morchella seasoning and its formula is as follows: Morchella mycelium pellets 80%, pepper 5%, dried mandarin peel 5%, cinnamon 5%, fennel 3% and clove 2%. Wei et al. [63] dried and crushed the fermented *Ganoderma lucidum* mycelium, and then mixed it with flour and cassava starch. This can be used to produce puffed food with health functions.

#### 3.1.2. Flavor in Beverages

The extracellular liquid from the liquid fermentation of edible and medicinal fungi also contains a variety of aromatic substances, such as alcohols, ketones, esters, etc., that can be added to food and beverages as natural flavors. Gao [64] explored the mixed fermentation process of *Ganoderma lucidum* with Monascus and black-tea fungus, and developed a functional black tea rich in γ-aminobutyric acid (GABA) and exopolysaccharide. Zhang et al. [65] mixed *Ganoderma lucidum* mycelium fermentation broth and distiller’s yeast with glutinous rice, and produced a sweet and sour *Ganoderma lucidum* glutinous rice wine through re-fermentation. Fungi Perfecti Company (USA) developed a coffee substitute MUD/WTR solid drink with functions for increasing vitality through mixing the mycelium of *Inonotus obliquus*, *Cordyceps militaris*, *Hericium erinaceus* and *Ganoderma lucidum* with other ingredients.

### 3.2. Functional Food Fields

The active products and by-products in fermented mycelium and fermentation broth can be used to develop functional foods, enrich food types and improve the nutritional value of foods.

The common functional foods developed from fermentation products are health drinks, wine, health oral liquids, *Hericium erinaceus* granules, *Ginseng tremella* pulp and so on. It was found that [66], by adding 5% mushroom mycelium instead of wheat to bread, there was no obvious change in rheology, texture and sensory factors, while high concentrations of ergothioneine and γ-aminobutyric acid could be produced after baking. Ran et al. [67] used *Ganoderma lucidum* mycelium polysaccharide extract as raw materials to prepare a flavored fermented milk (yogurt) beverage. Yang Qianqian [68] developed a snow pear-*Ganoderma lucidum* fermented functional beverage that is rich in polysaccharides, triterpenes, flavonoids and other nutrients, and it has certain antioxidant effects. A Morchella polysaccharide oral liquid developed by Fu [62] (Morchella polysaccharide extract 69.0%, chrysanthemum juice 25.9%, honey 5.2%) has certain effects on relieving physical fatigue and helps to reduce blood lipids. In addition, trace elements, such as selenium, zinc and iodine, can be enriched and converted into an organic state through liquid fermentation technology, thus developing functional foods [69,70,71]. Sun et al. [72] enriched selenium through the submerged fermentation of *Ganoderma lucidum* and obtained *Ganoderma lucidum* mycelium with selenium content of 2936 μg/g, which was used as the starting material to prepare a functional milk powder rich in *Ganoderma lucidum* polysaccharides and organic selenium. Myco Technology Company (Aurora, CO, USA) developed a bitter blocking agent called Clear Taste with mushroom mycelium as a raw material that can cover up the bitter taste and astringency in food, and can be used to reduce sugar and salt as a dietary supplement.

### 3.3. Health Food Field

Edible and medicinal fungi health food is one of the most active development areas in health foods. *Ganoderma lucidum*, *Lentinus edodes* and *Poria cocos* are the most common raw materials in the production of health products. The functions of edible fungi health food mainly include: enhancing immunity, relieving physical fatigue, improving sleep, assisting in protecting from chemical liver injury, assisting in lipid-lowering, etc. [7,73]. However, the most commonly used raw materials in health foods developed from edible and medicinal fungi are still the fruiting bodies, and mycelium is rarely used. Studies have shown that the active ingredients and medicinal value of *Cordyceps mycelium* obtained through fermentation are comparable to those of wild Cordyceps. Among them, fermented polysaccharides have a good regulatory effect on the nervous system and arrhythmia [74], and have been developed into products with an enhanced immune function, such as Cordyceps capsules and Cordyceps mycelium oral liquid. At present, health products developed from edible and medicinal fungi mycelium on the market are usually in the form of capsules and oral liquids such as *Ganoderma lucidum* mycelium capsules and *Lentinus edodes* mycelium liquid. Wang [75] investigated the liver protective effect of liquid fermented *Agaricus blazei Murrill* on alcoholic liver injured mice, and acquired the preparation technology and quality standard for the *Agaricus blazei Murrill* liver protective capsule.

### 3.4. Other Food Areas

#### 3.4.1. Food Preservatives

Studies have shown that edible and medicinal fungal polysaccharides can be used for food preservation, and they are often sprayed on the surface of food to isolate the external environment to maintain freshness. Lu et al. [76] coated apples with 2–6% *Inonus obliquus* polysaccharides, which can obviously reduce the weight loss rate, decay rate and respiration intensity of apples and maintain the hardness and freshness. Once *Penaeus vannamei* was soaked with different concentrations of Tremella polysaccharides, the sensory and blackening of shrimp meat were investigated [77]. The results showed that Tremella polysaccharides can inhibit the deterioration of the sensory quality and body surface blackening of South American white shrimp, and can effectively inhibit bacterial proliferation, and 0.6% Tremella polysaccharides have the best freshness maintaining effect. Sun [78] (CN201710852249.9) sprayed one or more of a *Ganoderma lucidum* polysaccharide solution, *Cordyceps militaris* polysaccharide solution and *Hericium erinaceus* polysaccharide solution on fresh meat, and the results showed that the freshness of the meat was prolonged and the storage quality was improved.

#### 3.4.2. Simulated Meat Products

With the increase in the world’s population, animal protein is facing the problem of insufficient supply, and artificial meat—simulated meat products with energy savings, emission reductions and rich nutrition—has come to light. According to the statistics, the scale of the plant-based artificial meat industry has reached USD 5 billion [79], but vegetable protein often lacks some essential amino acids, which leads to its low protein quality. The mycelium of edible and medicinal fungi contains all essential amino acids and some aromatic amino acids, and it also has a low-fat content. At the same time, the monosodium glutamate in mushrooms gives a taste similar to the amino acids in meat [80]. Fermented mycelium as a raw material for the production of simulated meat products instead of traditional animal protein will be one of the important development directions for health foods in the future [81]. Kim et al. [82] found that compared with soybean protein, artificial meat made from *Agaricus bisporus* mycelium has a better texture, hardness, elasticity and chewiness, as well as delicious taste characteristics that soybean protein does not have.

#### 3.4.3. Animal Feed

The research showed that the fermented mycelium from edible and medicinal fungi can also be used in animal feed. Yuan et al. [12] observed the culture effect of *Trichoderma sinensis* as a feed additive on South American white shrimp. The results showed that after 30 days of culture, the survival rate, daily gain rate and daily growth rate of shrimp had increased by 52%, 30% and 25%, respectively, and the immune function of the shrimp was also improved to some extent. Zhang et al. [83] investigated the immune-potentiating effects of the fermentation products of *Inonus obliquus* at vaccination in chickens. The results showed that the fermentation products of *Inonus obliquus* possessed significant immune-potentiating properties in chickens and could be a more economical and convenient oral adjuvant to improve vaccination in avian species.

## 4. Discussion

Edible and medicinal fungi are known for their high nutritional value. However, due to variations in strains, production areas, climate, cultivation of raw materials, cultivation techniques, production and processing, preservation and other factors, the functional components and contents of wild-collected and artificially cultivated fruiting bodies can vary significantly. Among the different cultivation methods, the liquid submerged culture of edible and medicinal fungi provides the best growth environment for mycelium by controlling the strain, culture substrate and environment. This method results in rapid mycelial growth and can directly regulate the production of high-yield active components. Moreover, several studies have demonstrated that the nutritional components of mycelium are similar to those present in the fruiting bodies. Therefore, liquid fermentation technology has broad application prospects for the industrial production of edible and medicinal fungi. Future research on the liquid fermentation of edible and medicinal fungi can focus on two aspects. First, optimizing the production process, including strain selection, culture conditions and fermentation parameters to improve the quality and yield of the active components. Second, studying the metabolic pathways of active components to identify key genes and enzymes that dictate their biosynthesis, which can help in the targeted design of fungal strains with higher production efficiency. In conclusion, liquid fermentation technology has great potential for the industrial production of fermented products from edible and medicinal fungi, and further research can unlock its full potential.

### 4.1. Breakthrough in the Liquid Fermentation Technology Bottleneck concerning Food and Drug Fungi

Future breakthroughs in the liquid fermentation technology of edible and medicinal mushrooms can focus on high-quality strains and large-scale fermentation tank culture. According to the current research situation, there are still some bottlenecks in the liquid fermentation technology of edible and medicinal mushrooms that urgently need to be solved.

An important direction to improve the economic benefits of the liquid fermentation of edible and medicinal fungi, is to obtain high-quality strains that meet the requirements of industrial production through breeding technology. Through mutagenesis, protoplast fusion and genetic engineering, the existing strains can be modified to change their nutritional value, flavor, appearance and color. At present, most of the strains used in the fermentation industry of edible and medicinal fungi are derived from cultivated strains, and their adaptability and stability vary widely, making their industrial production and application extremely unstable. In addition, the selection of varieties is particularly important, and the further development of mycelium as a raw material will be subject to the price competition of the fruiting bodies on the market. Therefore, mushrooms that are difficult to collect in the wild and cultivated artificially, or mushrooms with high economic value metabolites should be selected. For example, rare edible fungi, such as *Cordyceps sinensis,* are scarce in the wild and their artificial cultivation conditions are immature. Mycelium, with the active ingredient cordycepin, produced through liquid fermentation technology, compensates for the lack of resources. At present, the industrialized health food of *Cordyceps sinensis* mycelium has entered thousands of households.

The further development of mycelium from edible and medicinal fungi requires its large-scale growth in a bioreactor, and the yield of physiological active substances obtained is the same or better than that from a small-scale test. The current bioreactor is not completely suitable for the growth of edible and medicinal fungi mycelium. In order to develop a bioreactor suitable for edible fungi, it is necessary to have a deep understanding of the kinetics and growth physiology of their submerged fermentation process. However, with the development of computer and detection technology, image analysis systems and microelectrodes have been widely used in fermentation research that can detect important fermentation parameters in real time to clearly reflect the growth mode, influencing factors and physiological and biochemical processes of fungi in fermenters. As these studies continue, we can optimize the fermentation conditions and design suitable fermentation equipment for the growth of edible fungal mycelium in the future.

### 4.2. Expansion of the Application Field of Liquid Fermented Products from Edible and Medicinal Fungi

At present, the submerged fermentation products from edible and medicinal fungi are mainly used in the development of food/feed, medicine and daily chemical products in addition to strain preparation. In the field of food, MUD/WTR, a functional solid drink with improved vitality has been developed; in the field of medicines, and Bailing capsules (*Cordyceps militaris* powder) can relieve chronic kidney disease, Yunxing capsules (*Coriolus versicolor* powder) and *Hericium erinaceus* tablets *(Hericium erinaceus* mycelium) can treat chronic hepatitis. In the field of daily necessities, a variety of mycelium extracts have been added to cosmetics (such as *Ganoderma lucidum* mask) and care products for use in whitening and anti-aging applications. However, due to the limited application fields for mycelium at present, the products with fruiting bodies as raw materials are more common in the market.

The existing studies on the submerged fermentation of edible and medicinal fungi generally take mycelial biomass, total sugar and total triterpene yield as indicators, but the extraction degree of active functional factors is still insufficient. At present, the processing of edible and medicinal fungi after harvesting in China is still dominated by simple primary processing. While China is a big country in the production of edible and medicinal fungi, there is still a big gap between China and other countries in the processing and application area of edible and medicinal fungi. The future development of edible and medicinal fungi should focus on functional foods and health foods with high added value. Firstly, we could focus on the screening of excellent varieties of edible and medicinal fungi for pharmacological activities, expand new ideas for the development of the edible and medicinal fungi industry, and study in depth to identify the metabolic synthesis pathways of active substances from edible and medicinal fungi, so as to provide a new opportunity for industrial development. Secondly, we could focus on the extraction and development of functional factors in edible and medicinal fungi. For example, we should further explore the development of functional proteins with immunity, antioxidant, antibacterial and anti-viral activities, and enrich special flavoring substances, such as glutamic acid, aspartic acid and related nucleotides, to better replace meat products [84,85]. Last but not least, by using fermentation control agents from edible and medicinal fungi, the yield of active substances can be improved, and at the same time, the stable active substances can be provided by optimizing the separation technology of the fermentation products.

The long-term development trend of the edible fungi industry in the future is to develop targeted functional foods and health foods, and make advanced health drinks or pre-packaged convenience foods, so as to effectively change the traditional appearance of the edible fungi industry. At present, in China’s health product market, the products based on the active function of edible and medicinal fungi mainly focus on improving immunity, with few product types, narrow applicable population and serious homogenization. With the further approval of the application standards of mycelium in food, enterprises should also utilize various high and new technologies, such as vacuum frying technology and extrusion technology, to develop new raw materials and new formulas, and produce diversified products, such as children’s biscuits, health products and vegetarian meat for the elderly, that meet different age groups and different consumption needs.

## Figures and Tables

**Table 1:** Nutrient composition difference between the fruiting bodies and fermented mycelia from different edible and medicinal fungi (g/100 g dry weight).

Variety	Origin	Polysaccharide	Triterpenes	Amino Acid	Crude Protein	Protein	Fat	Fiber	Ash	Total Sugar	References
*Ganoderma leucocotextum*	Mycelia	1.54	0.03	-	-	-	-	-	-	-	[17]
	Fruiting body	0.79–0.87	0.07–0.09	-	-	-	-	-	-	-	
*Ganoderma* sp.	M	5.43	-	25.57	-	2.16	-	-	-	-	[33]
	F	1.55	-	8.61	-	0.60	-	-	-	-	
*Lyophyllum decastes*	M	1.77	-	-	28.30	-	2.78	5.32	6.06	54.7	[34,35]
	F	3.55	-	-	21.40	-	1.44	9.52	13.6	53.04	
*Ganoderma lucidum*	M	-	-	-	27.42	19.22	8.12	1.06	6.98	30.54	[36]
	F	-	-	-	8.88	6.22	6.6	18.10	5.54	22.34	
*Ganoderma sinense*	M	-	-	-	30.02	21.04	8.34	1.94	4.26	29.46	
	F	-	-	-	16.39	11.48	7.80	14.6	3.70	17.00	
*Ganoderma lucidum* (Chuanzhi no.6)	M	-	-	-	29.95	20.99	9.77	1.22	11.44	22.60	
	F	-	-	-	15.66	10.97	8.60	13.70	3.09	19.60	
*Grifola frondosa*	M	-	-	-	21.70	-	2.53	10.34	6.05	57.20	[37]
	F	-	-	-	31.50	-	1.70	10.70	6.41	49.70	
*Dictyophora indusiate*	M	-	-	-	24.82	-	2.09	6.60	-	51.50	[38]
	F	-	-	-	17.87	-	0.63	11.47	-	54.98	
*Cordyceps militaris*	M	3.44	-	17.65	-	-	-	-	-	-	[39]
	F	2.75	-	18.71	-	-	-	-	-	-	
*Volvariella bombycina*	M	-	-	0.44		0.35	0.36	1.11	0.35	6.30	[40]
	F	-	-	12.10		22.55	1.88	11.41	11.62	58.60	
*Fomitopsis pinicola*	M	-	-	43.89		45.76	2.84	0.70	4.76	42.36	[41]
	F	-	-	1.53–3.81		4.71–7.06	14.50–16.80	1.04–1.09	0.50–1.42	70.83–72.97	

Note: “-” means not detected.

**Table 2:** Comparison of the bioactivity differences between the fruiting bodies and fermented mycelia from different edible and medicinal fungi.

Variety	Origin	Component	Differences	Bioactivity	References
*Ganoderma lucidum*	Mycelia (M)	Polysaccharide content 3.81%	Monosaccharide composition: glucose, galactose; Mw: 1.412 × 10^4^ Da	Stimulating macrophage RAW264.7 to release NO	[54]
	Fruiting body (F)	Polysaccharide content 0.59%	Monosaccharide composition: glucose; Mw: 1.423 × 10^4^ Da, 1.153 × 10^4^ Da		
*Ganoderma lucidum*	M	Water extract	Hydrogen peroxide scavenging rate 80.2%	Antioxidant	[55]
	F	Water extract	Hydroxyl radical scavenging rate 62.9%		
*Sanghuangporous sanghuang*	M	Organic solvent extract	DPPH radical and ABTS^+^ radical scavenging rate corelated with total flavonoid content, the antioxidant activity is better than fruit body	Antioxidant and anti-tumor activity	[56]
	F	Organic solvent extract	Have a better anti-tumor activity on inhibiting HepG2 cells		
*Pleurotus eryngii*	M	Protein purity 68.3%	Better solubility and emulsifying activity 48%	Functional properties of proteins	[57]
	F	Protein purity 61.85%	Better water holding capacity and foaming property		
*Cordyceps militaris*	M	Organic solvent extract	DPPH radical scavenging rate (ORAC value) 10.39 μmol/g	Antioxidant activity	[58]
	F	Organic solvent extract	DPPH radical scavenging rate (ORAC value) 6.21 μmol/g		

**Table 3:** Food application of liquid fermented products from edible and medicinal mushrooms.

Application	Products	References
Common food	Mushroom sauce, condiments, expending foods, solid beverages	[59,60,61,62,63,64,65]
Functional food	Health drinks, fermented drinks, selenium-enriched foods	[66,67,68,69,70,71,72]
Health food	Health oral liquids, mycelium capsules	[73,74,75]
Others	Meat analogs, animal feed, food preservatives	[76,77,78,79,80,81,82,83]

## Data Availability

The data presented in this study are available on request from the corresponding author.

## References

[1] You M.L. (2007). The food Chinese (medicine) uses the fungus fermentation engineering research progress. Microbiology.

[2] Wu F., Zhou L.W., Yang Z.L., Bau T., Li T.H., Dai Y.C. (2019). Resource diversity of Chinese macrofungi: Edible, medicinal and poisonous species. Fungal Divers..

[3] Wei T., Zhang C.S., Chen Q.H., Zhou Y.P., Tian C.E. (2022). Liquid fermentation of *Ganoderma* and application of its products. Microbiology.

[4] Fazenda M.L., Seviour R., McNeil B., Harvey L.M. (2008). Submerged culture fermentation of “higher fungi”: The macrofungi. Adv. Appl. Microbiol..

[5] Zhao P., Guan M., Tang W., Walayat N., Ding Y., Liu J. (2023). Structural diversity, fermentation production, bioactivities and applications of triterpenoids from several common medicinal fungi: Recent advances and future perspectives. Fitoterapia.

[6] Guo S., Wang H.J. (2013). Submerged fermentation technology of edible fungus and its application. J. Shanxi Agric. Sci..

[7] Georgios B., Angeliki P., Petros K., Haralambos S. (2021). Recent trends in submerged cultivation of mushrooms and their application as a source of nutraceuticals and food additives. Future Foods.

[8] Humfeld H., Sugihara T.F. (1949). Mushroom mycelium production by submerged propagation. Food Technol..

[9] Kerezoudi E.N., Mitsou E.K., Gioti K., Terzi E., Avgousti I., Panagiotou A., Koutrotsios G., Zervakis G.I., Mountzouris K.C., Tenta R. (2021). Fermentation of *Pleurotus ostreatus* and *Ganoderma lucidum* mushrooms and their extracts by the gut microbiota of healthy and osteopenic women: Potential prebiotic effect and impact of mushroom fermentation products on human osteoblasts. Food Funct..

[10] Xie C., Tang P., Yan S., Yang Q., Zhang Z., Gong W., Zhu Z., Zhou Y., Yan L., Hu Z. (2020). Comparative study on bioactivities from Lingzhi or Reishi medicinal mushroom, *Ganoderma lucidum* (Agaricomycetes), gives an insight into the fermentation broth showing greater antioxidative activities. Int. J. Med. Mushrooms.

[11] Chi J.L., Li X., Wang Y.H., Fan L.J. (2021). Research status and prospect of *Cordyceps militaries* feed additive. J. Microbiol..

[12] Yuan H.M., Lin J.M., Zou J.R., Yu L.J., Zheng Y.B. (2022). Effects of Hirsutella sinensis as feed additive for *Penaeus vannamei*. Fujian J. Anim. Husb. Vet. Med..

[13] Shanghai Health Bureau (1978). Hougujun Pian. Zhongcaoyao Tongxun.

[14] Saddam S., Fazal U., Muhammad N., Muhammad Y., Asma A., Sajid A., Wajid Z. (2022). Mentha:Nutriional and health attributes to treat various ailments including cardiovascular diseases. Molecules.

[15] Sana A., Arshad F., Rameesha A., Mujeeb U.R., Walaa F.A., Majid A., Abdulhakeem S.A., Syed M.B.A., Daniel I.H., Saddam S. (2022). Identification, biochemical characterization, and safety attributes of locally isolated *Lactobacillus fermentum* from *Bubalus bubalis* (buffalo) milk as a probiotic. Microorganisms.

[16] Hsu T.H., Shiao L.H., Hsieh C.Y., Chang D.M. (2022). A comparison of the chemical composition and bioactive ingredients of the Chinese medicinal mushroom DongChongXiaCao, its counterfeit and mimic, and fermented mycelium of *Cordyceps sinensis*. Food Chem..

[17] Liu Y.F., Tang Q.J., Wang J.Y., Li C.H., Feng N., Wang C.G., Tang C.H., Zhang J.S. (2021). Comparison of bioactive components in fruiting body and fermented mycelium of *Ganoderma leucocontextum*. Acta Edulis Fungi.

[18] Zhu X.L., Yue Y.W., Zhang J.S., Feng J., Tang Q.J., Feng N., Han W. (2020). NMR attribution and bioactivity evaluation of a triterpene in mycelia of *Ganoderma lingzhi*. Mycosystema.

[19] Zhao B.J., Guo Y.B. (2022). Advances in extraction, purification and bioactivity of polysaccharides from edible fungi. China Biotechnol..

[20] Jing X.Y., Mao D.B., Geng L.J., Xu C.P. (2013). Medium optimization, molecular characterization, and bioactivity of exopolysaccharides from *Pleurotus eryngii*. Arch. Microbiol..

[21] Assis I.S., Chaves M.B., Silveira M.L.L., Gern R.M.M., Wisbeck E., Furigo A., Furlan S.A. (2013). Production of bioactive compounds with antitumor activity against sarcoma 180 by *Pleurotus sajor-caju*. J. Med. Food.

[22] Luo Q., Xu J.W. (2021). Monosaccharide composition and antioxidant activity of extracellular polysaccharides and intracellular polysaccharides in liquid ferment of *Ganoderma lucidum*. Acta Edulis Fungi.

[23] Du B., Yang Y.D., Bian Z.X., Xu B.J. (2017). Characterization and anti-inflammatory potential of an exopolysaccharide from submerged mycelial culture of *Schizophyllum commune*. Front. Pharmacol..

[24] Xu H., Chen L.L., Zhao S.Z., Qiu J.Y., Sun H., Xin X. (2016). Recent progress in anti-tumor activities of ganoderma triterpene from the mycelia of liquid-cultured ganoderma lucidum. J. Qilu Univ. Technol..

[25] Cai C.S. (2018). Extraction and Activity Analysis of Total Triterpenoids from Mycelium of *Sanhuang porus sanghuang* Liquid Fermentation. Master’s Thesis.

[26] Zhou X., Zhuang S.Y., Li Y.J., Liu R.Y., Liu C.M., Li S.N., Zhang Y.C. (2022). Extraction, screening and mass spectrometry analysis of activie triterpenoid components in *Ganoderma lucidum*. Mod. Food Sci. Technol..

[27] Ahlborn J., Stephan A., Meckel T., Maheshwari G., Zom H. (2019). Upcycling of food industry side streams by basidiomycetes for production of a vegan protein source. Int. J. Recycl. Org. Waste Agric..

[28] Kurbanoglu E.B., Algur O.F., Zulkadir A. (2004). Submerged production of edible mushroom *Agaricus bisporus* mycelium in ram horn hydrolysate. Ind. Crops Prod..

[29] Rahgo Z., Samadlouie H.R., Mojerlou S., Jahanbin K. (2019). Statistical optimization of culture conditions for protein production by a newly isolated *Morchella fluvialis*. BioMed Res. Int..

[30] Gang J., Liu H., Liu Y.H. (2016). Optimization of liquid fermentation conditions and protein nutrition evaluation of *Mycelium* from the caterpillar medicinal mushroom, *Cordyceps militaris* (Ascomycetes). Int. J. Med. Mushrooms.

[31] Bishop K.S., Kao C.H.J., Xu Y.Y., Glucina M.P., Paterson R.R.M., Ferguson L.R. (2015). From 2000years of *Ganoderma lucidum* to recent developments in nutraceuticals. Phytochemistry.

[32] Baby S., Johnson A.J., Govindan B. (2015). Secondary metabolites from Ganoderma. Phytochemistry.

[33] Zhang L.Y., Xiong X.H., Shen C., Shen E.G. (1998). Comparative study on products of fruiting body and submerged fermentation of *Ganoderma* sp.. Edible Fungi China.

[34] Xi Y.L., Mao A.L., Wang X.Q., Gui J.H., Wang Z.J., Wei S.L. (2010). Assessment for protein nutrition of fruit bodies, mycelia and fermentation broth of *Lyophyllum decastes*. Mycosystema.

[35] Xi Y.L., Wang Z.J., Wang X.Q., Wei S.L. (2010). Comparative analysis of nutrients in fruit body, mycelia and fermentation broth of *Lyophyllum decastes*. Food Sci..

[36] Zhou X.W., Lin J., Zhou L. (1998). Determine and analysis on primary nutritional components of *Ganoderma* spp.. J. Shaanxi Norm. Univ. (Nat. Sci. Ed.).

[37] Sun P.L., Xuan Y.W., Qiu J.P. (2001). Fermentation and composition analysis of *Grifola frondosa* mycelium. Edible Fungi.

[38] Ma Y.H., Zhang F.Y. (2004). Determination of the nutritive components of mycelia and fruitbody of Dctyophora Indusiata. J. Shanxi Agric. Univ..

[39] Zhu L.N., Gao X.H., Liu Y.F., Zhang H.X., Zhou S., Zhang Z., Li C.H., Tang Q.J. (2019). Effect of culture method on the active components in *Cordyceps militaris*. Acta Agric. Shanghai.

[40] Chen Y.X., Qin C., Zhang S.S., Xi Y.L., Wei S.L. (2021). Analysis of nutrient content comparison of fruiting body, mycelium and fermentation broth of *Volvariella bombycine*. Edible Fungi China.

[41] Zhou P.C., Zhang L.M., Cui Y., Song J.X., Nie L.R., Zheng Z.Q., Lu J.K., Kang C.C., Han P.P., Jia S.R. (2021). Comparative analysis of nutrients in frtuiting bodies and cultured mycelia of *Fomitopsis pinicola*. Food Sci..

[42] Gao H., Gu W.Y., Ding X.L. (2006). Studies on antitumor activity of ethanol extracts from liquid-cultured mycelium of *Agaricus brasiliensis* sp.. J. Food Sci. Biotechnol..

[43] Huang J., Li A.X., Zhou X., Wang S.M. (2016). The immune function and anti-tumor effects of *Ganoderma tsugae* fermentation product on H22 liver ascites tumor mice. J. Pharm. Res..

[44] Fijakowska A., Muszyńska B., Sukowska-Ziaja K., Kala K., Pawlik A., Stefaniuk D., Matuszewska A., Piska K., Pekala E., Kaczmarczyk P. (2020). Medicinal potential of mycelium and fruiting bodies of an arboreal mushroom *Fomitopsis officinalis* in therapy of lifestyle diseases. Sci. Rep..

[45] Yu X.L. (2014). Study of the Liquid Fermentation of *Tricholoma matsutatk* and the Anti-Tumor Activity of Polysaccharides. Master’s Thesis.

[46] Li P.Z., Zhang K.C. (2000). Isolation, purification and bioactivities of exopolysaccharides from fermentated broth of *Ganoderma lucidum*. Acta Microbiol. Sin..

[47] Li Z.L., Li X.X. (1994). Isolation, purification and identification of schizophyllan. Mycosystema.

[48] Li Z.M. (2012). The Research on Submerged Fermentation vs. Extracted and Purified of Polysaccharide and Biological Activity of *Pholiota dinghuensis bi*. Master’s Thesis.

[49] Carrieri R., Manco R., Sapio D., Lannaccone A., Fulgione A., Papaianni M., Falco B.D., Grauso L., Tarantino P., Lanniello F. (2017). Structural data and immunomodulatory properties of a water-soluble heteroglycan extracted from the mycelium of an Italian isolate of *Ganoderma lucidum*. Nat. Prod. Res..

[50] Hu W.J., Song M.K., Wang C.Y., Guo Z., Li Y., Wang D. (2021). Structural characterization of polysaccharide purified from *Hericium erinaceus* fermented mycelium and its pharmacological basis for application in Alzheimer’s disease: Oxidative stress related calcium homeostasis. Int. J. Biol. Macromol..

[51] Liu Y.T. (2021). Study on the Effect of Different Liquid Fermentation Conditions on the Active Polysaccharides from *Inonotus obliquus*. Master’s Thesis.

[52] Zhang Q., Wu C.E. (2016). Physicochemical properties and antioxidant activities of protein from *Morchella esculenta* mycelium. Acta Agric. Zhejiangensis.

[53] Dou H.J., Sun L.H., Guo W.T. (2015). Antibacterial activity of mushroom fermentation broth to drug-resistant bacteria. China Brew..

[54] Yu H.Z., Liu Y.F., Zhou S., Yan M.Q., Xue L., Tang Q.J. (2016). Comparison of the polysaccharides from fruiting bodies, mycelia and spore powder of *Ganoderma lingzhi*. Mycosystema.

[55] Cai M.T., Tang Q.J., Wang Y.L., Huang Y.W., Feng J., Feng N., Yu L. (2018). Comparison on antioxidant activity of spore, fruiting body and mycelium of *Ganoderma lucidum*. Acta Edulis Fungi.

[56] Zhang J.F., Zhang Z., Wang W.H., Zhang H.N., Feng N., Wu D., Liu Y.F., Yang Y. (2020). Comparison of active secondary metabolites between mycelia and fruiting bodies of *Sanghuangporous sanghuang*. Mycosystema.

[57] Chen Y.F., Bao D.P., Chen H.Y., Feng J., Luo Y., Zhang X., Zou G., Zhao Y. (2021). Extraction and functional properties of proteins in Pleurotus eryngii mycelium from submerged liquid fermentation. Acta Edulis Fungi.

[58] Guo J.K., Yu X.W., Liu S.T., Rao P.F., Wu S.B., Zhao S.L., Qiao J.H., Chen R.C., Luo M.X. (2022). Antioxidant properties of powders of *Cordyceps militaris* fungus, sporophore and mycelium and their effects on body surface electrical potential difference along acupuncture meridians. J. Food Sci. Technol..

[59] Dermiki M., Phaphensophon N., Mottram D.S., Methven L. (2013). Contributions of non-volatile and volatile compounds to the umami taste and overall flavour of shiitake mushroom extracts and their application as flavour enhancers in cooked minced meat. Food Chem..

[60] Rathore H., Prasad S., Kapri M., Tiwari A., Sharma S. (2019). Medicinal importance of mushroom mycelium: Mechanisms and applications. J. Funct. Foods.

[61] Wu X.T. (2014). Molecular Identification and Liquid Fermentation of *Tricholoma mongolicum* and Application of Mycelium Metabolite. Master’s Thesis.

[62] Fu T.Y. (2013). Study of *Morchella esculenta* Functional Food Based on the Submerged Fermentation. Master’s Thesis.

[63] Wei Y.F., Wang L.S., Qin F.Z. (2009). Development of *Ganoderma lucidum*-fermented instant food with high-quality dietary fiber. Food Sci..

[64] Gao J.J. (2013). Study on the Mixed Fermentation Technics of Blace Tea by *Ganoderma lucidum Monascus* and *Kombucha*. Master’s Thesis.

[65] Zhang S., Wang J., Cheng H. (2018). Development of fermented glutinous rice wine with *Ganoderma lucidum* mycelia. Food Ind..

[66] Ulziijargal E., Yang J.H., Lin L.Y., Chen C.P., Mau J.L. (2013). Quality of bread supplemented with mushroom mycelia. Food Chem..

[67] Ran J.S., Wang H.C., Chen J.Z., Ran M.C., Chen C. (2018). Preparation of fermented milk beverage from *Ganoderma lucidum* mycelia and *Ginkgo biloba* leaves. Farm Prod. Process..

[68] Yang Q.Q. (2022). Study on the Preparation Technology for a New Type of Snow Pear Beverage from *Ganoderma lucidum* Fermented Snow Pear Juice. Master’s Thesis.

[69] Poursaeid N., Azadbakht A., Balali G.R. (2015). Improvement of zinc bioaccumulation and biomass yield in the mycelia and fruiting bodies of *Pleurotus florida* cultured on liquid media. Appl. Biochem. Biotechnol..

[70] De Souza D.F., Da Silva M.C.S., De Souza T.C., Rocha G.C., Kasuya M.C.M., Eller M.R. (2023). Effect of selenium-enriched substrate on the chemical composition, mineral bioavailability, and yield of edible mushrooms. Biol. Trace Elem. Res..

[71] Wang H., Zhang Q.L., Zuo J.J., Yan G.Y., Huo L.G. (2015). Study on zinc-rich ability of Hyphae of *Pleurotus nebrodensis* in submerged culture. Chin. Agric. Sci. Bull..

[72] Sun Z.T., Wang H.Z., Yan Y.C., Yao L.T. (2003). Study on technology of functional powered milk made from seenriched *Ganoderma lucidum* mycelia. China Dairy Ind..

[73] Su Y.W., Lu P., Guo Q.Q., Wang J. (2016). Progress in edible fungus by submerged fermentation. J. Food Saf. Qual..

[74] Song Y.Q., Wang L., Guo S.P. (2008). Research progress of *Cordyceps sinensis* sall and its mycelia. SCI-TECH Inf. Dev. Econ..

[75] Wang H. (2015). Study on Liquid Fermentation of *Agaricus blazei murill* and Preparation of Protecting Liver Capsule. Master’s Thesis.

[76] Lu Y., Li J.G., Meng N., Sun Y., Gao Y.J. (2011). Effect of *Inonotus obliquus* polysaccharide on preservation of apple. Food Mach..

[77] Deng W.J., Qian L., Zhang J., Zhang Z.J., Zhang Y.N. (2021). Effects of *Tremella fuciformis* polysaccharides on *Penaeus vannamei* qualities during storage. Storage Process.

[78] Sun Y.G. (2017). Fresh Meat Preservation Method.

[79] Zhang G., Zhao X., Li X., Du G., Zhou J., Chen J. (2020). Challenges and possibilities for bio-manufacturing cultured meat. Trends Food Sci. Technol..

[80] Sun L.B., Zhang Z.Y., Xin G., Sun B.X., Bao X.J., Wei Y.Y., Zhao X.M., Xu H.R. (2020). Advances in umami taste and aroma of edible mushrooms. Trends Food Sci. Technol..

[81] Gu K.F., Zhou C.Y., Li X.B. (2017). Nutritive and medicinal value edible fungi. Food Ind..

[82] Kim K., Choi B., Lee I., Lee H., Kwon S., Oh K., Kim A.Y. (2011). Bioproduction of mushroom mycelium of *Agaricus bisporus* by commercial submerged fermentation for the production of meat analogue. J. Sci. Food Agric..

[83] Zhang L., Lin D., Li H., Yu S., Bai J., Ding Z., Wu J. (2018). Immunopotentiating effect of *Inonotus obliquus* fermentation products administered at vaccination in chickens. Mol. Cell. Probes.

[84] Mau J.L. (2005). The umami taste of edible and medicinal mushrooms. Int. J. Med. Mushrooms.

[85] González A., Cruz M., Losoya C., Nobre C., Loredo A., Rodríguez R., Contreras J., Belmares R. (2020). Edible mushrooms as a novel protein source for functional foods. Food Funct..

